
JOURNAL INFORMATION
==============================
NLM Title Abbreviation: Atlanta Med Surg J (1884)
NLM Journal ID: 101251623

Atlanta Medical and Surgical Journal (1884)

Publisher: Biomedical Journal Digitization Project

ARTICLE INFORMATION
==============================
PMCID: PMC9201853
PMID: 35829605
Article version: 1
Subjects: Original Communications

The Diagnosis of Pregnancy* An address delivered before the Atlanta Society of Medicine.

Hardon Virgil O. M.D. Atlanta, Ga.

Print publication date: 1899 Feb
Volume: 15
Volume Series: New Ser
Issue: 12
First page: 798
Last page: 803
License: This work is free of known copyright restrictions. For more information, please see PMC Back Issue Digitization.
License URL: https://creativecommons.org/publicdomain/mark/1.0/

==============================

### THE DIAGNOSIS OF PREGNANCY.*

By VIRGIL O. HARDON, M.D.,
ATLANTA, GA.

The subject upon which I shall speak to-night is an old one. It is as old as the human race. It is probable that Eve, when pregnant with Abel, availed herself of the experience of her previous pregnancy in making a diagnosis of her condition. Or if we adopt the Darwinian theory we may reasonably assume that our simian ancestors, those anthropoid apes whose blood is supposed to course through the veins of all of us, made use of their recently developed intellectual faculties to determine the meaning of the symptoms which arose when conception occurred. From that time to the present this question has agitated the minds of pregnant women and of men whose business it is to care for pregnant women. Since the question has thus been studied for six thousand years, or six million years, as the case may be, it would seem presumptuous in me to appear before you to-night with the expectation of saying anything new. But even though all has been said that can be said, it is sometimes profitable for us to pause and sum up our knowledge, to take account of stock as it were, in order to see where we stand. It is my purpose to thus sum up our present knowledge of the diagnosis of pregnancy and, perhaps, to elicit some discussion on the subject which shall bring out the views of other men and correct my own, where they shall prove erroneous.

This subject is one which interests the whole profession. It is not the specialist alone who is concerned in it. Even the oculist has occasion to treat albuminuric retinitis due to pregnancy, and the dermatologist sees cases of chloasma uterina arising from the same cause. In the majority of cases the physician is not called upon to make an early diagnosis of pregnancy. Women who have borne children before are sufficiently familiar with the symptoms to be able to recognize the condition when it

*An address delivered before the Atlanta Society of Medicine.

arises, and even women who are totally inexperienced in such matters are usually content to wait until the symptoms become unmistakable. Nor is it in the later months of pregnancy that we are called upon to make a diagnosis, when the patient has reached the fifth or sixth month, when her abdomen has enlarged to such an extent that her condition is obvious to every passer-by, and when the fetus has foretold its advent by leaping in its mother's womb. There is no need for the physician to confirm the diagnosis which the mother has already made for herself. But when the timid, shrinking, tearful maiden, who has loved "not wisely, but too well," comes to us with a story of having missed her menses from taking cold and hopes against hope that by an examination we may confirm her diagnosis of the causation of her amenorrhea, it is then that our decision assumes vital importance. Or when the newly married bride who has been looking forward to a year or more of social enjoyment finds her prospects of pleasure thwarted by the prospect of pregnancy, then, also, our opinion is eagerly sought. Or when an inheritance of an estate, or a title, or, possibly, even a crown, hangs upon the question of pregnancy, then, again, the question is a momentous one.

Since the diagnosis of pregnancy presents no difficulties after the early months have passed, I shall confine my discussion to those symptoms which present themselves at a time when the question may be considered a doubtful one. The first symptom, as a rule, which attracts attention and which leads to a suspicion of pregnancy is the cessation of menstruation. This is looked upon by the laity as strong evidence of a pregnant condition, and rightly so. When a woman has been regular in her menstruation all her life, and when this function suddenly ceases, especially if she be a woman who has a right to be pregnant, this symptom furnishes strong presumptive evidence, and yet it has its fallacies from two points of view. In the first place, women not infrequently menstruate after conception has taken place, and it is a matter of common observation, which I have frequently had occasion to verify in my practice, that when conception takes place just before the menstrual period, that period occurs, though in a modified form, being diminished in quantity and duration. Not only may menstruation occur a single time immediately after conception has taken place, but it may occur once, twice, thrice, or even more. Cases are reported in which menstruation has persisted all during pregnancy. I have always been somewhat skeptical in regard to such cases, being inclined to attribute the flow of blood to other causes. On the other hand, women frequently fail to menstruate who are not pregnant. Anemia, depressing chronic diseases of any kind, any condition where the loss of blood is interfered with by nature as a conservative process, may cause menstruation to fail to appear. Depressing emotions, such as grief, and even a fear of pregnancy in a woman who has no right to be pregnant, may cause this symptom. Cessation of the menses, therefore, cannot be considered an evidence of pregnancy except in the presence of other corroborative indications.

A second early symptom of pregnancy, which is of value in making a diagnosis, is the so-called morning sickness. The term is a misnomer, because in my experience the nausea of pregnancy occurs as frequently at other times of the day, or even of the night, as in the morning. This is by no means a conclusive sign of pregnancy, since nausea closely similar to it may occur in other conditions. Reflex disturbances of the stomach may arise from a diseased condition of the womb, as well as of other organs. Yet, in the majority of cases, the nausea of pregnancy is characteristic in the fact that it is not accompanied by constitutional disturbances. There is, however, one diseased condition which may give rise to a nausea so closely simulating that of pregnancy as to be easily mistaken for it. I refer to the nausea which accompanies uremia. The distinction between the two is, of course, to be made by an examination of the urine. The nausea of pregnancy occurs in some cases, if we may judge from the statements of physicians, as early as the first week. As a rule, however, it is not until after a suspicion of pregnancy has been excited by the failure of menstruation that the nausea makes its appearance. In the majority of cases the nausea consists simply of a discomfort, which, though severe, entails no serious consequences. The patient is able, in spite of the nausea, to retain sufficient nourishment to support life. There is, however, a class of cases in which the nausea assumes, from the very beginning, an entirely different type. It is accompanied by so great an amount of physical and mental depression, such rapid impairment of strength, with the development of such high temperature and so feeble and rapid a pulse, that the picture presented by it is characteristic. This form of nausea is distinctly pernicious in type and tends to a fatal result. It is in this class of cases that interruption of pregnancy becomes necessary in order to save life. It is this class of cases, also, which so closely simulate nausea from uremic poisoning. These cases, fortunately, are rare. Since nausea simulating that of pregnancy may arise from other causes, it is obvious that this symptom is not a conclusive one. It is only when taken in connection with other symptoms that it is of value. In the absence of other symptoms it can furnish no positive evidence.

The symptoms which I have referred to are subjective symptoms which can be discovered by the patient herself and which lead her to suppose that conception has taken place. There is another group of symptoms, objective in character, which appear early in pregnancy, and which are of as great, if not greater, value than the subjective ones. The first of these is the change in the consistency of the neck of the womb by which it loses its normal hardness and becomes soft and compressible. The well-known saying of Goodell is doubtless familiar to all of you. He says: "If the cervix is hard like your nose, the woman is not pregnant. If it is soft like your lips, she is pregnant." This symptom can be detected as early as the beginning of the second month, and is, in my judgment, the most valuable of the early signs. Another symptom, which can be discovered by examination in the early months, is the change in the color of the mucous membrane of the vagina and the cervix. The mucous membrane loses its normal rosy, pink tint, and becomes changed to a dark, dusky purple. This change is due, of course, to the physiological congestion produced by pregnancy. This congestion extends not only to the deeper blood-vessels, but to the capillaries which course through the superficial layers of the mucous membrane. The change of color is most pronounced in the mucous membrane of the vestibule, but can be detected over the whole vagina and the cervix.

It is much more marked in some cases than in others, but I have never seen a pregnant woman in whom it could not be detected after the first month. Its constancy makes it, therefore, a valuable indication of pregnancy, but its value is somewhat impaired by the fact that a similar discoloration may arise from any diseased condition which produces conjestion of the pelvic blood-vessels. I have frequently seen it accompanying fibroid tumors. It is not, therefore, a conclusive sign, but is of great value as corroborative evidence.

Another class of signs which develop early in pregnancy are the changes which take place in the breasts. As early as the end of the first month, and sometimes even earlier, the breasts become turgid and enlarged, the areola around the nipple becomes deeper in color and is elevated above the surrounding surface of the breast. The superficial veins become visible and can be traced as blue lines in the skin. Yet these changes may occur when pregnancy does not exist. I have seen them all, combined, moreover, with the presence of milk in the breast, in a woman who had never been pregnant.

There are several other signs which have more or less value as corroborative evidence of the existence of pregnancy, but their value is slight, either because they develop late in pregnancy, or because by themselves they have little significance. Those which I have mentioned are the ones upon which reliance is to be placed in making a diagnosis. No one of them is conclusive, and it is only when two or more are associated together that we can make even an approach to a positive diagnosis. Even although all be present, a diagnosis cannot be absolutely certain, for there are conditions other than pregnancy in which all of them may exist. We are forced, therefore, to the conclusion that in the early months of pregnancy it is impossible to make an absolutely positive diagnosis; that is to say, a diagnosis that we would be willing to swear to in a court of law. Yet when several of the signs are grouped together, the picture is sufficiently complete to enable us to assure the patient of a strong probability of pregnancy. Where momentous issues are at stake, however, it is safer for us to reserve our judgment and await further developments. Fortunately it is not often that a positive diagnosis becomes necessary in the early months, and by the time later symptoms establish the diagnosis beyond a doubt, the woman will already have made a diagnosis for herself. It is, therefore, my rule during the first three months of pregnancy, when consulted by a patient for the purpose of determining this point, to reserve my opinion, to avoid committing myself, and to wait for time to render a verdict from which there can be no appeal.

---
